# Adult PFAPA - a single centre experience

**DOI:** 10.1186/1546-0096-13-S1-P176

**Published:** 2015-09-28

**Authors:** O Donnelly, T Youngstein, R Pepper, D Rowczenio, P Hawkins, H Lachmann

**Affiliations:** 1 Royal Free Hospital London NHS Foundation Trust, London, UK; 2 University College London, UK National Amyloidosis Centre, London, UK; 3 University College London, Nephrology, London, UK

## Introduction

Variant PFAPA affecting adults has been previously reported but appears to be either extremely rare or systematically underdiagnosed.

## Objectives

To retrospectively analyse patients with symptoms resembling PFAPA seen in an adult fevers clinic in the UK.

## Patients and methods

Patients were sought from the UK National Amyloidosis Centre database using PFAPA, fever, pharyngitis, lymphadenopathy, and aphthous ulcers as search terms. Data were collected on demographics, symptoms, investigations and treatment.

## Results

15 patients were identified. 13 were male and all of white European origin. None gave a family history of similar symptoms. Current median age is 28.3 years with median symptom duration of 15 years. 6 patients presented after the age of 16, and 5 before the age 5. 3 patients reported precipitants for their attacks, in all cases stress and fatigue. 13 patients reported regular attacks every 4-6 weeks. Fever was present in 100%; cervical lymphadenopathy in 93%, pharyngitis in 73%; oral aphthous ulceration in 40%; abdominal pain in one third, rash and red eyes in 13%. 13 of 15 patients reported at least 3 of fever, lymphadenopathy, pharyngitis or aphthous ulceration with attacks.

Sequencing of MEFV, MVK, TNFRSF1A was normal in all cases. 7 patients provided samples during attacks with a median CRP 27 mg/L and SAA 205 mg/L. All 15 had normal inflammatory markers when well.

47% underwent tonsillectomy without lasting benefit in any case. Corticosteroids had been used by 60% with 4 good responses and 4 partial responses; 4 patients continue on intermittent prednisolone. 14 (93%) have tried colchicine with 2 complete, 4 good and 6 partial responses and 12 (86% of exposed) remain on long term prophylaxis. One patient received anakinra and and 3 tried cimetidine with little effect.

All patients achieved heights and weights within the normal adult range. 13 of 15 are either in full time education or employment. No patients have developed AA amyloidosis.

## Conclusion

Variant PFAPA is seen in adults. In our series 40% presented after the age of 16 and 33% presented in the typical age range of less than 5 years with persistent symptoms into adulthood. Compared to typical childhood PFAPA symptoms seem very similar but more patients are refractory to conventional treatment with corticosteroids or tonsillectomy. Colchicine given as long term prophylaxis is the most effective treatment although complete responses are rare. Despite ongoing symptoms and elevated CRP and SAA with attacks no patients have severe social or physical consequences of their disease.

